# Effect of one week of yoga on function and severity in rheumatoid arthritis

**DOI:** 10.1186/1756-0500-4-118

**Published:** 2011-04-12

**Authors:** Shirley Telles, Kalkuni V Naveen, Vaishali Gaur, Acharya Balkrishna

**Affiliations:** 1 Patanjali Research Foundation, Patanjali Yogpeeth, Haridwar, India

## Abstract

**Background:**

Previous studies have shown that yoga practice improved the hand grip strength in patients with rheumatoid arthritis (RA).

**Findings:**

Sixty-four participants with RA (group average age ± S.D., 46.5 ± 9.6 years; 47 females) were assessed at the beginning and end of a one week yoga program. The Stanford Health Assessment Questionnaire (HAQ), hand grip strength, rheumatoid factor, and C-reactive protein levels were assessed on the first and last day and compared using a t-test for paired data. All participants showed reduced disability scores of the HAQ and rheumatoid factor levels, with an increase in bilateral hand grip strength in male participants alone.

**Conclusions:**

This single group study indicated that a brief intensive yoga program was beneficial in RA, with decreased disability, better functionality and changes in the rheumatoid factor levels suggesting improvement.

## Background

A randomized controlled trial studied the effect of yoga on twenty patients with RA [1]. While conventional measurements (e.g., ring size, duration of morning stiffness, grip strength, and the HAQ) were carried out, a significant difference between groups was seen in the left hand grip strength alone.

In a later study a combination of yoga techniques also improved hand grip strength which was the only assessment taken [2]. To our knowledge no study has simultaneously monitored disability and pain, hand grip strength, as well as the rheumatoid factor levels and C Reactive Protein simultaneously in patients with rheumatoid arthritis following yoga. A population-based rheumatoid arthritis incidence cohort (1955-1995, aged > or = 18 years) was followed up longitudinally until January 1, 2006 or until death [3]. Patients who were rheumatoid factor positive had higher than expected mortality from cardiovascular and respiratory diseases. Hence the present trial was planned to assess the short term impact of yoga on the rheumatoid factor levels and other variables.

## Materials and methods

Sixty-four participants (forty-seven females) were selected from one hundred participants who had enrolled for a one week yoga program. The selected participants had a diagnosis of active RA based on the criteria of the American College of Rheumatology [4]. All participants were on non-steroidal anti-inflammatory drugs, and fifteen were taking corticosteroids in doses of 10 mg of prednisolone (or equivalent) every day. The doses were kept stable during the trial though participants were asked to consult their rheumatologist or a doctor at the yoga center at the end of the week to review their medication. Their ages ranged between 20 and 70 years (group mean ± S.D, 46.5 ± 9.6 years). All participants were right hand dominant based on a standard handedness inventory. The signed informed consent was obtained from all the participants before they took part in the study. The study had the approval of the Institution's Ethical Committee.

The trial was a single group trial, with assessments before and after the one week intervention. None of the patients dropped out of the trial.

All participants were administered the HAQ [5]. Hand grip strength was measured using a standard method [6] and a hydraulic hand grip dynamometer (Lafayette Instrument, Model TKK 5401, U.S.A). Each hand was tested in a single trial and the order of testing each hand was alternated for different patients. Serum rheumatoid factor and C-reactive protein levels were measured by immunoturbidometry [7].

The one week residential yoga camp had two sessions each day. The first session was between 05:00 hours and 07:30 hours and the second session was between 17:00 hours and 19:30 hours. Each day participants practiced voluntarily regulated yoga breathing (called *pranayamas *in Sanskrit; 50 percent of the time), loosening exercises (*sukshma vyayama; *25 percent of the time which included flexion, extension and rotation of the shoulder and wrists, and flexion and extention of the elbow and fingers combined with slow and deep breathing), and yoga postures (*asanas; *25 percent of the time). Some of the movements are similar to those used in a study on yoga for carpal tunnel syndrome [8]. The breathing techniques included high frequency yoga breathing at the rate of 1.0 Hz (*kapalabhati*), breathing through alternate nostrils (*anulom-vilom pranayama*), exhalation with specific sounds (*brahmari *and *udgeeth pranayamas*), and breathing with a period of breath holding or with a voluntarily partially constricted glottis (*bahya *and *ujjayi pranayamas*, respectively).

Data recorded on Day 1 and Day 7 of the residential yoga camp were compared with the t- test for paired data using SPSS Version 18.0. Data were analyzed for the group as a whole (n = 64), as well as for female (n = 47) and male (n = 17) participants separately.

## Results

The group as a whole showed a significant decrease in the Disability Index on the last day compared to the first (p < 0.01), particularly related to eating (p < 0.05) and common daily activities (p < 0.01). For female participants there was an improvement in dressing (p < 0.001), arising (p < 0.001) and walking (p < 0.001). For male participants the improvement was in dressing (p < 0.05), arising (p < 0.001), walking (p < 0.05), and in grip (p < 0.001). There was no significant change in the level of pain (p > 0.05 in all cases).

The percentage change in (a) disability scores of the HAQ, (b) rheumatoid factor levels, (c) left hand grip strength, and (d) right hand grip strength were calculated. Here percentage change was {[Post/Pre*100]-100}. (2) The percentage changes (a, b, c, d) mentioned above [1] were correlated with (i) age and (ii) severity of RA using the Pearson correlation test for correlations with age and the Spearman correlation test for correlations with severity. No linear correlation was found with the percentage changes and either age or severity of RA. However this does not mean that these factors did not influence the outcome.

Male participants alone showed a significant increase in the bilateral hand grip strength. The rheumatoid factor was significantly lower on the last day compared to the first for the group as a whole (p < 0.05, one-tailed).

For all variables, the groups mean values ± S.D. are given in Table 1.

**Table 1:** Assessments in patients with rheumatoid arthritis before and after yoga

Variable	Assessment	Whole group	Males	Females
Health Assessment Questionnaire (HAQ) Total score	Before	0.85 ± 0.50	0.56 ± 0.32	1.03 ± 0.52
	
	After	0.58 ± 0.40*	0.04 ± 0.08*	0.16 ± 0.12*

Pain scale of the HAQ	Before	6.29 ± 2.31	5.53 ± 2.31	6.74 ± 2.22
	
	After	5.22 ± 2.17	4.59 ± 2.31	5.58 ± 2.14

Hand grip strength Right hand (Kg)	Before	21.75 ± 9.42	27.28 ± 11.46	19.08 ± 5.96
	
	After	23.53 ± 8.71*	27.79 ± 7.18*	18.05 ± 5.45*

Hand grip strength Left hand (Kg)	Before	21.66 ± 7.77	28.82 ± 10.44	20.87 ± 5.67
	
	After	23.57 ± 7.35	29.63 ± 6.29	19.85 ± 5.09

Rheumatoid factor (IU/mL)	Before	57.33 ± 134.93	4.44 ± 2.99	73 ± 150.62
	
	After	52 ± 126.82**†**	3.55 ± 2.26	67.56 ± 1.41

C-Reactive protein (mg/L)	Before	7.79 ± 19.35	4.49 ± 5.87	8.77 ± 21.82
	
	After	7.31 ± 9.60	10.40 ± 12.21	6.39 ± 18.84

Values are group mean ± S.D.

*p < 0.05, After yoga compared to Before, t-test for paired data, two-tailed

**† **p < 0.05, After yoga compared to Before, t-test for paired data, one- tailed

## Discussion

A one week yoga program reduced the disability index scores and decreased the rheumatoid factor in participants of both sexes, while male participants alone showed an increase in bilateral hand grip strength. While no linear correlation was found with the percentage changes and either age or severity of RA, this does not mean that these factors did not influence the outcome.

The improvement in the participants' HAQ Scores, with a decrease in the Disability Index after a week of yoga practice is of importance as it is known that a decrease in disability improves functionality [9]. The other main benefit following yoga was the improvement in hand grip strength though this was limited to male participants. The reason why female participants did not improve is not known.

The reduction in self-reported discomfort in the participants in the present study could be related to the stretching exercises which were part of the yoga program, as stretch reduces pain associated with working at a computer work station [10]. An earlier study speculated that increased hand grip strength after yoga practice could be due to reduced need for oxygen along with changes in the availability of energy and the oxidation of glucose [11]. However these are just speculations. The actual mechanisms have yet to be worked out.

At the start of the camp all participants tested positive for rheumatoid factor. The group as a whole had levels considerably higher than normal which is 20 IU/ml. This remained abnormally high even after yoga. Male patients had levels within the normal range which remained within the normal range after yoga. In contrast to this, female patients had abnormally high rheumatoid factor levels which reduced, but remained above normal after yoga. The reason for the gender difference is not clear. The different in baseline severity between males and females could be one of the reasons for the difference in outcome. The decrease in rheumatoid factor levels was considered a favorable outcome, not just because high rheumatoid factor levels are associated with poor functionality and greater disability, but also with higher mortality-risk [3].

In summary the present one week yoga program reduced the disability scores based on the HAQ, improved hand grip strength (in male participants), and reduced the rheumatoid factor levels in participants with RA, though levels remained above normal.

## Limitations

The main limitation of the study was the fact that this was a single group study without a control group who received no intervention (or an alternative intervention) for comparison. Also, the group was self-selected. All of them had chosen to join the one week yoga camp. This is of importance as it is now recognized that our feelings and beliefs can influence our behavioral responses [12]. Apart from this participants were in a residential facility. The conditions were not especially different from a normal home hence this factor is unlikely to have influenced the results. However the fact that participants were receiving a vegetarian diet may have influenced the outcome, as a vegetarian diet is known to be beneficial in participants with rheumatoid arthritis [13]. In fact the three limitations of the study are related. In a study like this, where participants voluntarily join for a specific intervention (in this case, yoga), most of them are unable to spare the time, and hence would be unwilling to be assigned to a wait-list control group. In a residential setting, factors such as a change in diet or a change of surroundings could favorably impact the findings. A separate trial would have to be planned on patients, who would be prospectively enrolled and randomized, whenever they attend an outpatient department, as has been done for other diseases [14]. Alternatively, long-stay inpatients undergoing conventional treatment in a regular hospital can also be randomized as yoga and control groups [15]. Either of these options would be considered for a further, randomized controlled trial, with a longer duration follow-up, in patients with rheumatoid arthritis.

## List of abbreviations

HAQ: Stanford Health Assessment Questionnaire; RA: Rheumatoid arthritis.

## Competing interests

The authors declare that they have no competing interests.

## Authors' contributions

ST designed the study, interpreted the data and compiled the manuscript. KVN supervised the selection of patients and data collection and helped in compiling the manuscript. VG collected the data and performed the statistical analysis. AB decided the details of the intervention. All authors (ST, KVN, VG and AB) have read and approved the final manuscript.
